# Temporomandibular joint biomechanics and equine incisor occlusal plane maintenance

**DOI:** 10.3389/fbioe.2023.1249316

**Published:** 2023-09-20

**Authors:** Tomas Rudolf Sterkenburgh, Bettina Hartl, Christian Peham, Michael Nowak, Michal Kyllar, Silvio Kau

**Affiliations:** ^1^ Polyclinic for Dental Preservation and Periodontology, University of Leipzig, Leipzig, Germany; ^2^ Department of Industrial Engineering, Business Administration and Statistics, DEGIN Doctoral Program, Universidad Politécnica de Madrid, Madrid, Spain; ^3^ Department of Pathobiology, Institute of Morphology, Vetmeduni Vienna, Vienna, Austria; ^4^ Department of Companion Animals and Horses, Movement Science Group, University Clinic for Horses, Vetmeduni Vienna, Vienna, Austria; ^5^ Veterinary Practice Dr. M. Nowak, Equine Clinic Meerbusch, Meerbusch, Germany

**Keywords:** equine dentistry, incisor occlusal surface, group function occlusion, dental wear, malocclusion, mastication, biomechanics

## Abstract

In equine dentistry, the physiological incisor occlusal surface is visually perceived as a plane with a distinct inclination to the head’s coronal plane, extending rostro-ventrally to caudo-dorsally. To better understand the formation of this inclined plane and its connection to dental wear, we investigated the hypothesis that it arises from masticatory movements and the considerable distance between mandibular articular heads and the incisor occlusal surfaces, acting as the three points of support for the mandibles. Leveraging data from a large-scale clinical study involving static and dynamic orthodontic measurements in horses, we approximated the mandibular movement range where incisor occlusion and dental wear occur. By introducing and testing a segment coordinate system, we explored possible angular deviations from the occlusal plane caused by mandibular roll and pitch rotations during two lateral mandibular movement patterns, protrusion and retrusion. Theoretical biomechanical calculations and simulations confirmed the visual perception of the incisor occlusal surface as a plane. To further examine our assumptions, we employed a simple mechanical simulator to assess incisor normal occlusion and provoked malocclusions (diagonal, smile, and frown bite) by modifying temporomandibular joint (TMJ) movement patterns. The results from clinical investigations were corroborated by both the theoretical analysis and mechanical simulations, strengthening our understanding of the biomechanical basis behind the physiological incisor occlusal plane maintenance in horses. These findings have significant implications for equine dental health and contribute to a thorough understanding of TMJ dynamics.

## Introduction

Equine dental wear is caused by attrition (tooth-to-tooth contact) and abrasion (tooth-to-food contact) (Imfeld, 1996). This dental wear occurs due to compressive loading when two tooth areas are pressed against each other by masticatory muscle force and due to shearing loads when these tooth areas are moved over each other while maintaining chewing pressure. Occlusion has a decisive influence on dental wear; therefore, small deviations in posture and occlusion can lead to chronic dental problems (Easley et al., 2011a; Taylor et al., 2018). Dental wear is influenced by extrinsic factors such as type of forage and abrasive particles in the food and intrinsic factors such as the hardness of the tooth structures and mastication biomechanics (Muylle et al., 1999).

In horses, chewing is a unilateral process that occurs on either the right or left side (Collinson, 1994; Tremaine, 1997; Baker and Easley, 2005; Staszyk et al., 2006a). The chewing cycle is described differently in the literature: during the opening stroke, the mandible moves ventrally (Bonin et al., 2006; Bonin et al., 2007; Simhofer et al., 2011), laterally either to the balancing (Bonin et al., 2006; Bonin et al., 2007; Sterkenburgh et al., 2022) or toward the working side (Bonin et al., 2006; Staszyk et al., 2022) and slightly rostrally (Simhofer et al., 2011; Staszyk et al., 2022) or caudally (Bonin et al., 2006; Bonin et al., 2007). The mandible either rotates around a vertical axis located symmetrically between the mandibular articular head (AH) of both sides (median axis) (Bonin et al., 2006) or it rotates around the AH of the working side mandible, while the AH of the balancing side slides in a rostral direction (Staszyk et al., 2022). A lateral movement may be superimposed. During the closing stroke, the mandible moves dorsally (Bonin et al., 2006; Bonin et al., 2007; Simhofer et al., 2011) until the mandibular cheek teeth of the working side contact those of the maxilla (Collinson, 1994; Tremaine, 1997; Baker and Easley, 2005). Furthermore, the mandible moves either rostrally (Bonin et al., 2006; Bonin et al., 2007) or caudally (Simhofer et al., 2011) and laterally to the working side (Bonin et al., 2006; Bonin et al., 2007; Sterkenburgh et al., 2022) during the closing stroke. The power stroke causes the mandibular cheek teeth to grind upon the maxillary teeth in a movement from ventral to dorsal and either in a medial (Tremaine, 1997; Bonin et al., 2006; Bonin et al., 2007; Sterkenburgh et al., 2022) or lateral (Simhofer et al., 2011) direction. Shortly before reaching the neutral position—at the point of maximum lateral displacement—the six mandibular and maxillary incisor teeth come into contact and masticatory pressure is carried by the incisor teeth and temporomandibular joints (TMJs). The contact between the incisor teeth at the end of each chewing cycle in the post-power “recovery” stroke is called incisor landing (Staszyk et al., 2022; Sterkenburgh et al., 2022). Throughout the chewing process, masticatory forces are distributed differently to the two TMJs, left and right maxillary and mandibular cheek tooth arcades, and mandibular and maxillary incisor teeth. The masticatory forces on the mandibular cheek teeth are highest during the power stroke, reaching up to 1956 N (Staszyk et al., 2006a; Huthmann et al., 2009). The bite force of the incisor teeth during food prehension is approximately 2% of body weight (Hongo and Akimoto, 2003). Chewing rates differ depending on forage, ranging between 68.5 and 83.9 chews/min (Weinert et al., 2020).

Occlusion of the incisor teeth is laterally restricted to the area between the two points of maximum lateral excursion to separation (LETS), also known as excursion to molar contact (EMC). In this area of incisor occlusion, masticatory pressure is mainly supported by the incisor teeth and the TMJs. To examine molar occlusion, the lateral excursion to molar contact test (LMC) is measured during equine dental examination (Limone et al., 2022). EMC ranges from 4.2 to 30 mm, with a mean of 11.7–12.3 mm and a median between 11 and 12 mm (Rucker, 2002; Rucker, 2004; Pimentel and Zoppa AL do, 2014). To identify LMC, the neutral position of the maxillary and mandibular incisor teeth relative to each other is first determined with the mouth closed. From this position, the lower jaw is maximally deflected to the left and right, while the mouth remains closed. At the point of maximum deflection, the cheek tooth arcades come into contact and push the incisor teeth apart because of their angle (Rucker, 2004). The distance of the neutral position, respectively, to the right and left maximum deflection points with the jaws closed is called the left or right LETS (Easley et al., 2011b). Occlusal abnormalities in incisor teeth are common, and the reported numbers vary from 48.6% to 60% of the horses examined (Pimentel and Zoppa AL do, 2014; Kunz et al., 2020). Smile bite or ventral curvature (CV) of the incisor arcade, frown bite or dorsal curvature (CD), and incisor slant or diagonal incisor malocclusion (DIM) have been reported in 21.4%, 17.1%, and 27%–48.7% of examined horses, respectively (Pellachin, 2013; Kunz et al., 2020).

Accounting for all variables when simulating tooth wear *in vitro* is difficult (Lambrechts et al., 2006). Karme et al. (2016) used equine cheek teeth fixed in an artificial mastication apparatus to determine differences in microwear patterns using four different feeds. Sterkenburgh et al. (2022) performed computer simulations that mimicked cheek teeth wear using a simplified two-dimensional model. The stress and strain energies in the periodontal ligament and alveolar bone of equine incisor teeth were analyzed using finite element analysis (Schrock et al., 2013). To date, no study has investigated incisal wear in horses.

We hypothesized that the physiological occlusal surface of equine incisor teeth is planar. The starting point was the evidence-based knowledge about the masticatory process in horses. From the clinical orthodontic measurements, we derived insights on the potential TMJ dynamics required for maintaining the occlusal plane of the incisor teeth through theoretical geometric and biomechanical considerations. Furthermore, we utilized a mechanical mastication simulator to prove our theoretical considerations and demonstrated that incisor malocclusions such as DIM, smile bite, and frown bite may result from specific TMJ/mandibular movement patterns.

## Materials and methods

### Clinical assessment of static and dynamic orthodontic parameters

**FIGURE 1:**
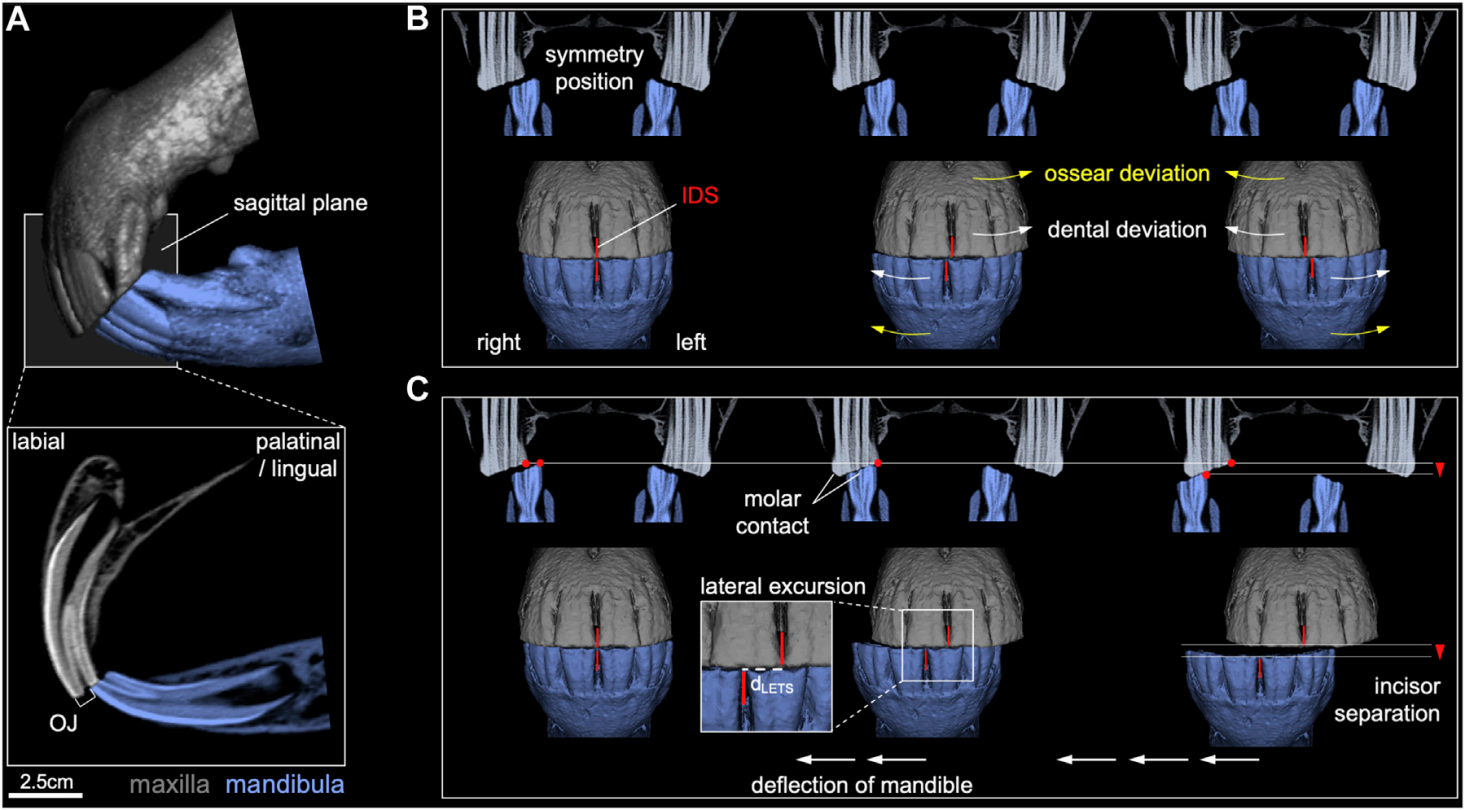
Graphical illustration of evaluated static and dynamic orthodontic parameters. **(A)** Overjet (OJ) for maxillary and mandibular central incisor horizontal occlusal plane discrepancy at the described head position. **(B)** Maxillary and mandibular central incisor interdental space (IDS) center offset. **(C)** Distance of mandibular lateral excursion to incisor separation (d_LETS_) and molar contact.

We analyzed the dental reports of 609 horses which have undergone a regular dental check (dental prophylaxis). The reports were collected by a single examiner (SK) over the period of April 2017 to September 2018 in Germany and Austria. The analyzed data included measurements of static and dynamic orthodontic parameters, i.e., horizontal overbite (also termed overjet (OJ)), horizontal underbite (also termed underjet (UJ)) and central incisor interdental space center offset (CO) at jaw symmetry position (static) and LETS (dynamic). Briefly, a detailed pre-treatment examination of the oral cavity was performed under sedation with 0.01 mg*kg^-1^ detomidine hydrochloride (Detogesic, Zoetis, Berlin, Germany) and 0.1 mg*kg^-1^ butorphanol (Torbugesic, Zoetis, Berlin, Germany). A dental holster (Pegasos4D, Waldkirch, Germany) was used to support the head during examination and orthodontic measurements. The head was elevated to a neutral position as previously outlined by Kau et al. (2020). Orthodontic measurements were performed using a stainless-steel length and angle measuring device (0–100 mm, Shinwa Rules, Niigata, Japan). OJ and UJ were measured as the distance between the mesio-occluso-labial edges of the maxillary and mandibular central incisor teeth (Figure 1A) (vertical deviation = 0 mm). This measure only considered horizontal over-/underbite with incisor teeth still in occlusion and occlusal planes oriented parallel. The center offset of the interdental spaces between maxillary and mandibular central incisor teeth was measured to assess functional asymmetry in the rostral dentition (Figure 1B), which was achieved by manually centering the maxillary and mandibular cheek teeth while visually controlling their alignment by deflecting the cheeks. The center offset distance to either side was considered the starting point for bilateral assessment of the incisor LETS (Figure 1C). Horses with a true vertical overbite or underbite, with maxillary and mandibular incisor’s occlusal surfaces not in contact, or with marked incisor malocclusions were excluded from the study. The data analyzed in this study were limited to measurements obtained before the dental treatment.

### Fundamentals and assumptions of mandibular and TMJ movement under incisor occlusion

**FIGURE 2:**
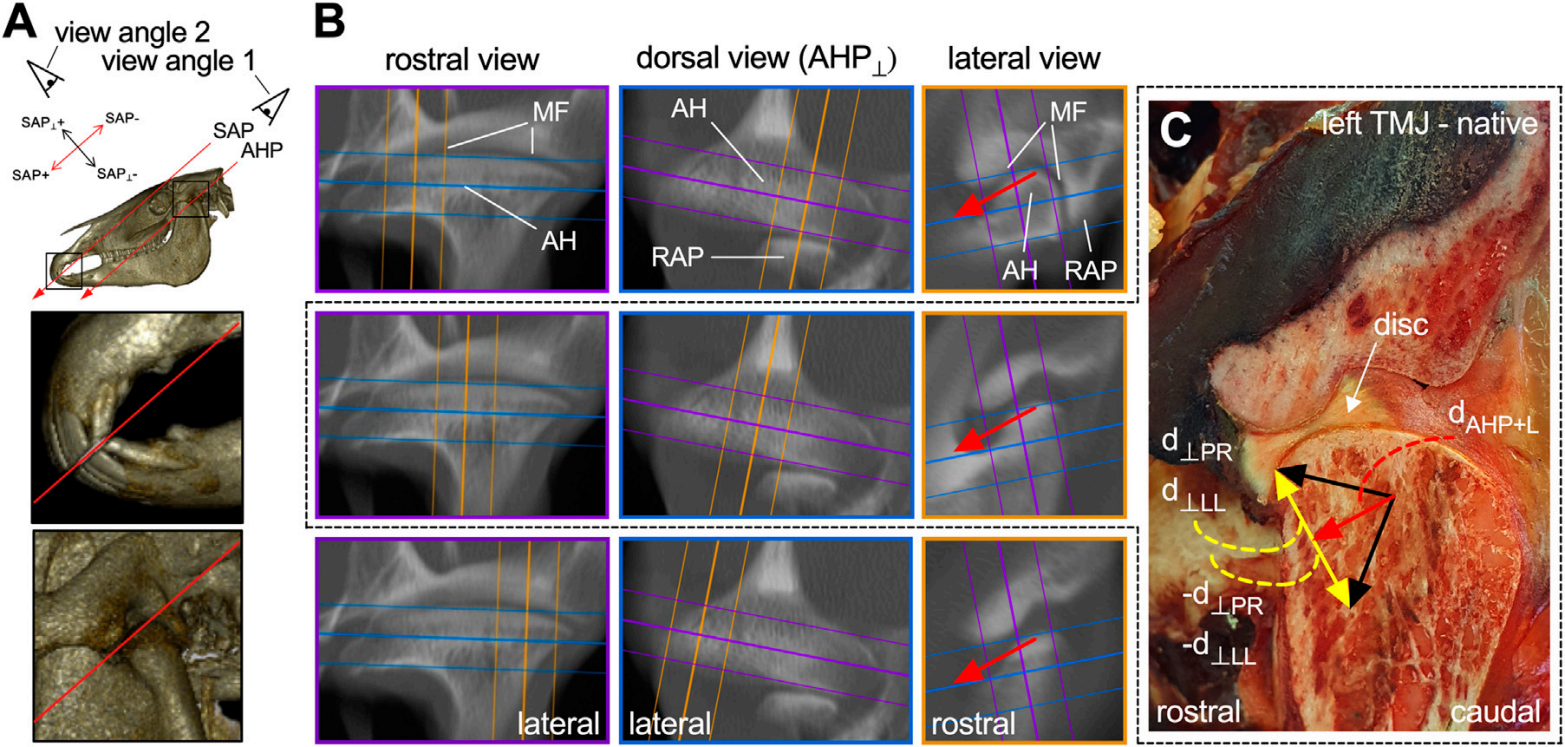
Multiple views of considered dentofacial planes and assumptive depiction of TMJ protrusion movement components. **(A)** Study view angle on the occlusal surface of incisor teeth sagittal angle plane (SAP) and mandibular articular head plane (AHP) in the TMJ. Image details point out SAP and AHP. **(B)** Multilevel multiplanar mean intensity projection reconstruction and native depiction of a left TMJ. Arrows indicate protrusion movement vectors. MF, mandibular fossa; AH, mandibular articular head; RAP, retroarticular process. **(C)** Native section at mid TMJ level; lateral view. Black arrows: Upward estimates of directional deviation with vector components in red and yellow. Vector naming as used for theoretical calculations, below (⊥ eq. perpendicular).

During incisor occlusion, the mandible rests on three spatially separated areas, i.e., two TMJs and incisor occlusal surfaces. The mandible and consequently the incisor teeth undergo translatory movements of protrusion (forward) and retrusion (backward) under occlusion in a caudo-dorsal to rostro-ventral direction within the sagittal angle plane (SAP). This term indicates that the surface plane angulation can be measured in a sagittal plane (Listmann et al., 2017) (Figure 2A). In the following text, the direction of mandibular protrusion under incisor occlusion was referred to as the “SAP+" direction and the corresponding retrusion direction as “SAP-“. A movement perpendicular to the SAP is referred to as the “SAP⊥+” (directed rostro–dorsally) or as “SAP⊥-” (directed caudo-ventrally) (Figure 2A). Based on anatomical considerations, we assume a main protrusion movement of mandibular articular heads also largely along the SAP+ direction, but in a parallel articular head plane (AHP) (Figures 2A–C). Retrusion under incisor occlusion in the opposite direction (SAP-) is likely limited by the retroarticular process (RAP) (Figures 2A,B). For movements of the mandibular AH, we apply the terminology accordingly, as “AHP+,” “AHP-,” “AHP⊥+,” and “AHP⊥-.” With these designations, we transferred our considerations to the segment coordinate system of the incisor occlusal surface plane, thus avoiding imprecise combinations such as caudo-dorsal and rostro-ventral, in the following text. Lateral movements were referred to as “lateral".

From anatomical observations, it appears likely that the protrusion movement of the mandibular AHs under incisor occlusion goes mainly in the SAP+ direction with minor deviations. Figure 2C confines a range of assumed protrusion movements as an upward estimate of the directional deviation from this SAP+ movement direction and mark the vector components accordingly.

This section further outlined the biomechanical dimensions to evaluate and model the effect of TMJ dynamics and the resulting mandibular motion on incisor occlusal surface wear and maintenance of the occlusal surface as a plane. The assumed TMJ dynamics were deduced from observations of static and dynamic orthodontic measurements of mandibular motion reported in the literature, from own measurements, and from theoretical assumptions thereof that are drawn from this study.

#### Mandibular range of motion under incisor occlusion

First, we determined the range of possible mandibular positions that would allow contact between the occlusal surfaces of the maxillary and mandibular incisor teeth and subsequent dental wear. This range primarily had a latero-lateral and SAP+ to SAP– direction as well as combinations of both and was estimated based on existing evidence and our own measurements during this study. LETS/EMC, often determined as part of standard dental care using the “lateral excursion test” (Figure 1C), describes the range of translational movement out of the symmetrical position of the mandible to the left and right while maintaining incisor occlusion. The magnitude of this displacement was reported in the literature as 1–30 mm with a mean average (±SD) of 12 ± 3.6 mm (Rucker, 2004; Pimentel and Zoppa AL do, 2014; DeLorey, 2007; Carmalt et al., 2003), and we also additionally evaluated this in our study. An SAP+ and SAP– translational jaw movements (i.e., protrusion and retrusion under incisor occlusion) changed dynamically during mastication and passively changes with different head positions (Griffin, 2013). The total magnitude for this displacement during mastication was given in the literature as 5–18 mm in adult horses and 3–4 mm in foals. Considering the reported values, the mean average (±SD) in adult horses was 8.8 ± 2.2 mm (Bonin et al., 2007; Simhofer et al., 2011). SAP+ and SAP– mandibular movement, however, can be influenced by cheek tooth pathologies, dental treatment, and feed consistency (Simhofer et al., 2011; Carmalt et al., 2003). The proportion of masticatory SAP+ and SAP– jaw movement under incisor occlusion can only be assumed. We measured the central OJ and UJ with an elevated head position, suggesting that a more orthognathic and full occlusion superimposed antagonistic incisor teeth with a lowered head. The latter occurs naturally during grazing and feeding. Regardless of the rostro-caudal mandibular displacement that occurs, it can be stated based on geometric considerations that incisor occlusion ends when the mandibular arcade is displaced in SAP+ or SAP– direction until the occlusal surfaces are no longer in contact. We also assumed that during jaw deflection under incisor occlusion, there may be minimal mandibular pitch and a roll around a latero-lateral and SAP “in-plane” axis, respectively. Since incisor occlusion may involve various directions of jaw movement, a comprehensive understanding of these movements, including translation to the TMJs, will aid in understanding equine incisor wear and occlusal plane maintenance.

#### Movement pattern of the TMJs under incisor occlusion

**FIGURE 3:**
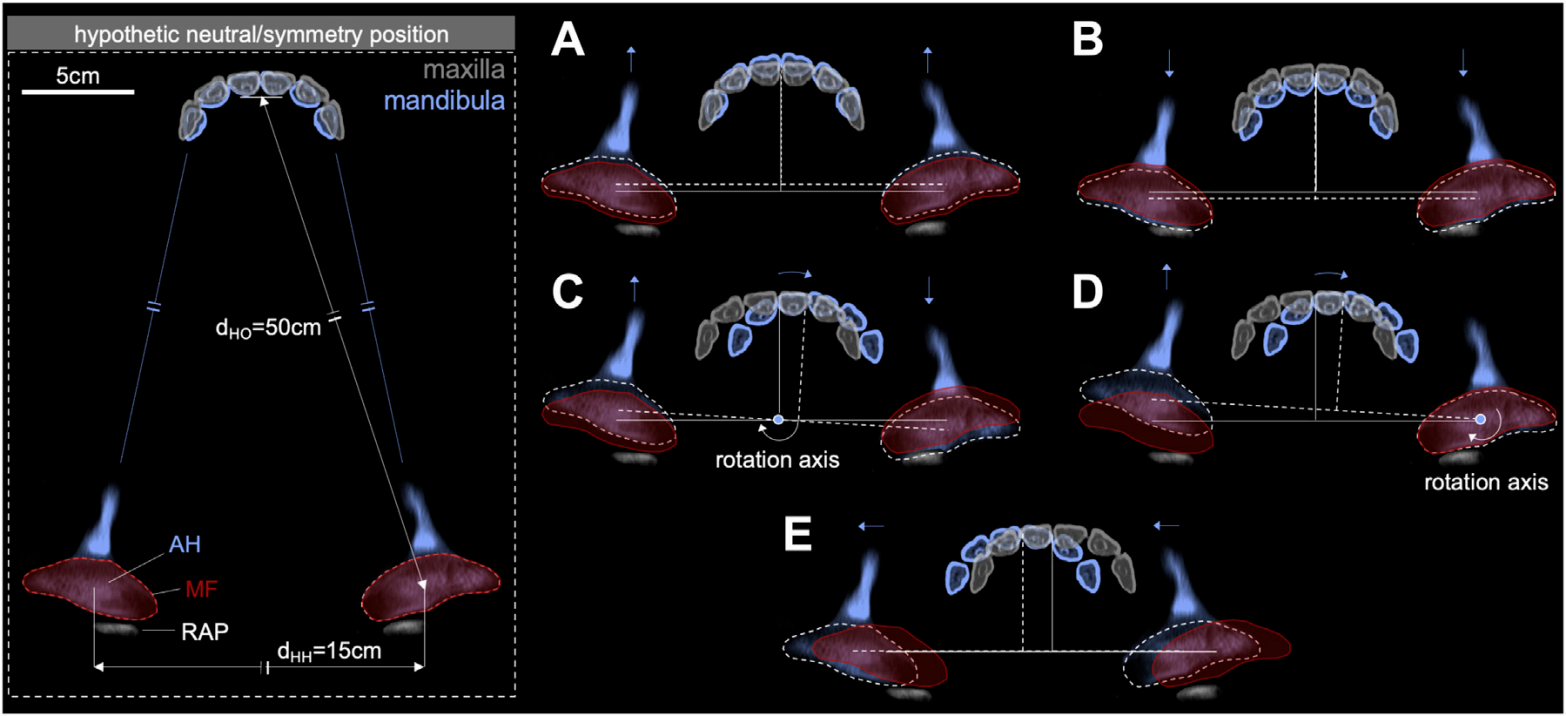
TMJ movements under incisor occlusion. **(A)** Protrusion (SAP+) and **(B)** retrusion (SAP–) range, **(C)** laterotrusion movement around an interarticular rotation axis along SAP⊥+ to SAP⊥– (pattern no. 1), **(D)** laterotrusion movement around a mandibular articular head rotation axis along SAP⊥+ to SAP⊥– (pattern no. 2), and **(E)** latero-lateral translatory mandibular movement (side shift). AH, mandibular articular head; MF, mandibular fossa; RAP, retroarticular process.

Based on possible mandibular movements under incisor occlusion, the corresponding movement dynamics of the TMJs can be approximated and modeled accordingly. Due to the rigid bony mandible, any movement of the occlusal surface of the mandibular incisor teeth, whether rotatory or translatory, is transferred to the corresponding TMJs. Protrusion (SAP+) and retrusion (SAP–) translatory movements result in a corresponding AHP+ and AHP– movements of both mandibular AHs in the TMJs (Figures 3A,B). In horses, latero-lateral mandibular incisor movements are described as the rotation of the mandible around an axis, directed from SAP⊥+ to SAP⊥–, i.e., perpendicular to the SAP plane. Two rotational movement patterns are differentiated:

• First, resulting from a rotation of the mandible around a virtual axis located symmetrically between the two mandibular AHs. This causes the working-side AH to move backward and the balancing-side AH to move forward on a circular path around the interarticular axis of rotation (Figure 3C) (Bonin et al., 2006). It is further referred to as movement pattern no. 1.
• Second, based on anatomical boundaries and prior research on non-midline jaw movements and associated TMJ dynamics in humans, we further propose and theoretically model an alternative rotation around an axis via the AH on the working side, while the AH on the balancing side slides off in an AHP + direction (Figure 3D). It is further referred to as pattern no. 2. In humans, this type of movement is described as both rotation around and translation along a virtual helical axis (Koolstra, 2002). Staszyk et al. (2022) suggested this type of movement in horses; however, this has not been validated yet.

Using our mechanical simulator, combinations of the previously described TMJ motion patterns were simulated and checked for their impact on the incisor occlusal surface plane maintenance.

• Additionally, in humans, the lateral movement of the mandibular incisor teeth involves not only the rotation of the mandible around the working-side AH but also a lateral translatory component, that can occur progressively (Bennett movement) or spontaneously (immediate side-shift). This movement displaces the entire mandible toward the working side (Bennett, 1908; Preiskel and Goldstein, 2021). In our study, we also considered that this type of TMJ movement occurs in horses (Figure 3E), but do not use it in simulator experiments.

#### Calculations

After considering all the movement patterns, we quantified the extent and possibility of each movement. By performing geometrical calculations, we aimed to provide a theoretical estimate of the impact of each of the five movement patterns in Figure 3 on the occlusal surface of incisor teeth. These calculations served to understand and further model the mechanics of jaw movement and its effects on the occlusal surfaces of the incisor teeth.

For our theoretical considerations, we assumed two horse heads. First, a male 12-year-old Warmblood horse (WBL) with a distance of d_HO_ = 50 cm between the mandibular AHs and central incisor’s occlusal surface and a distance of d_HH_ = 15 cm between the AHs (interarticular distance), as shown in Figure 3. Second, a pony head was considered, being d_HO_ = 29.1 cm and d_HH_ = 9.7 cm. These values are derived from computed tomography (Warmblood) and skull (pony) measurements.

A) LETS of incisor teeth

**FIGURE 4:**
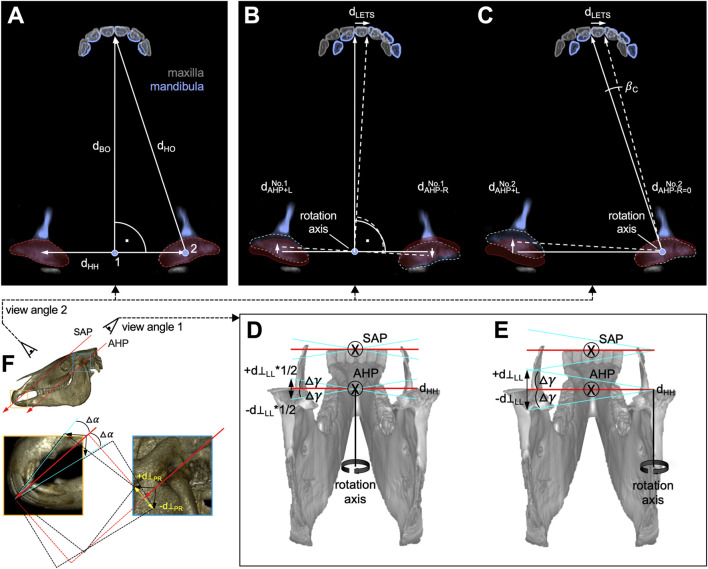
View on mandibular articular heads (AHs) and incisor teeth with distances, angles, and rotation axes in laterotrusion **(A–E)** and protrusion/retrusion **(F)** movement. **(A)** Neutral/symmetry position; 1, interarticular and 2, right AH SAP⊥ rotation axis. **(B, C)** Incisor and TMJ movements integrating rotation pattern no. 1 and no. 2. d_BO_, distance between interarticular “Bonin” pivot point and incisor occlusal surface; d_HO_, distance from AH to incisor occlusal surface; d_CC_, interarticular distance; d_LETS_, LETS distance; $d_{AHP+L}^{No.1}$ and $d_{AHP+L}^{No.2}$ left AH movement distances; $d_{AHP-R}^{No.1}$ , right AH caudal movement distance. **(D)** the perpendicular movement component d_⊥LL_ causes a roll around the SAP+ axis of Δγ. **(E)** For pattern no. 2, the perpendicular movement components are opposing + d_⊥LL_*1/2 and -d_⊥LL_*1/2. (**F**) Considerations to mandibular/incisor occlusal plane rotation (pitch) due to protrusion. No angular deviation occurred if the AH movement was parallel to the SAP+ direction (red arrows). A perpendicular movement component d_⊥PR_ caused a ∆α angle variance. The black arrows are upward estimates of the directional variances. SAP+, incisor occlusal surface protrusion direction; AHP + assumed AH plane protrusion direction.

First, the pivot point distance of pattern no. 1 to the incisor occlusal surface d_BO_ (Figure 4A) was calculated using the Pythagorean theorem as follows:

$$d_{BO}=\sqrt{d_{HO}^{2}-\frac{d_{HH}^{2}}{4}}.$$ (1)

Owing to the rotational movement, the lateral excursion represented a circle segment. With a small angle of rotation and a large distance between the center of rotation and incisor teeth in horses, a linear distance can be inferred. This corresponds to the length of the circular segment in a good approximation, which was followed in this study.

For pattern no. 1, the distance covered by the left (L) mandibular AH in AHP+ direction $d_{AHP+L}^{No.1}$ and the distance covered by the right (R) AH in AHP– direction $d_{AHP-R}^{No.1}$ are in correspondence to the lateral excursion $d_{LETS}$ of the six incisor teeth according to the theorem of intercept. $d_{LETS}$ , and the AH movement and the lateral excursion are related to each other via the length of the rotation arms:

$$d_{AHP+L}^{No.1}=-d_{AHP-R}^{No.1}=\frac{d_{HH}/2}{d_{BO}}*\;d_{LETS}.$$ (2)

For the alternative movement pattern no. 2, the AH of the right side has no translatory movement component, i.e., $d_{AHP-R}^{No.2}=0$ , while $d_{AHP+L}^{No.2}$ then results as follows:

$$\begin{array}{c} d_{AHP+L}^{No.2}=\frac{d_{HH}}{d_{HO}}*\;d_{LETS}\;∼{2*d}_{AHP+L}^{No.1}\\ with\;d_{HO}\;∼\;d_{BO} \end{array}.$$ (3)

To calculate the angular deviation from the occlusal plane in the incisor teeth based on distances, the possible rotation (roll) of the mandible (Figures 4D,E), occurring in connection with these movements, patterns was determined. If the rotation axis is perpendicular to SAP, no roll occurs. A motion component leaving this plane, however, will result in a deviation from the occlusal plane. The extent of the rotation is indicated by the motion component $d_{⊥LL}$ perpendicular to the plane in SAP⊥+ or SAP⊥– directions. Only if $d_{⊥LL}\neq 0$ , the latero-lateral motion was associated with roll and thus results an angular change.

Based on our anatomical observations, we assumed that both mandibular AH movement patterns no. 1 and no. 2 largely occurred in the described AHP plane.

For upward estimation of a maximum angle of rotation for movement pattern no. 2, we assumed a perpendicular movement component $d_{⊥LL}$ of the same size as $d_{AHP-R}^{No.2}$ , respectively, occurring perpendicular to the plane. This acted on the distance between the AHs (*d*_*HH*_) and produced a rotation (roll) of

$$∆\gamma =\mathrm{atan}\left(\frac{d_{⊥LL}}{d_{HH}}\right)=\mathrm{atan}\left(\frac{d_{AHP+L}}{d_{HH}}\right),$$ (4)

as described in Figure 4E. For movement pattern no. 1 (Figure 4D), we assumed an opposing ${1/2d}_{⊥LL}$ acting on $1/2d_{HH}$ , which leads to the same △γ.

For a better comprehension of the angular deviation △γ, which is due to lower jaw rolling motions, we look at the skull along the SAP+ and AHP+ axes with the eye position as marked in Figure 4F. This perspective is unusual but allows easier explanation of angular deviations (Figures 4D,E).

B) Protrusion under incisor occlusion

While excessive mandibular backward movement in AHP– direction is limited, forward movement at the level of incisor occlusal surfaces occurred along the occlusal surface plane in the SAP+ direction. The concomitant mandibular AH movement is in a similar direction (AHP+), however, followed AH mobility given by TMJ anatomy (Figures 2B,C). The different AH plane angles may have resulted from the shape of the articular surfaces. Angle variances may therefore lead to mandibular rotation (pitch) around the latero-lateral axis.

As an upward estimate in mandibular incisor forward movement (protrusion), we considered both mandibular AHs to have the same perpendicular movement component d_⊥PR_ in the SAP⊥+ or SAP⊥– directions. This results in an upward estimate for the deviation from the SAP+ direction as shown in Figure 4F. The angular change ∆α due to this movement was calculated as follows:

$$∆\alpha =\mathrm{atan}\left(\frac{d_{⊥\mathrm{PR}}}{d_{BO}}\right).$$ (5)

C) Lateral translatory mandibular excursion (immediate side shift)

During a lateral translatory movement of the entire mandible, it is to be expected that both AHs of the mandible show a movement component perpendicular to the incisor occlusal plane. Following the articular bone structure, the right and left perpendicular movement components likely not oppose but aim in SAP⊥– direction and differ in size. This would lead to mandibular roll, although there is no evidence that this translatory movement exists in horses.

### Mechanical simulation of TMJ movement and incisor occlusal surface wear

**FIGURE 5:**
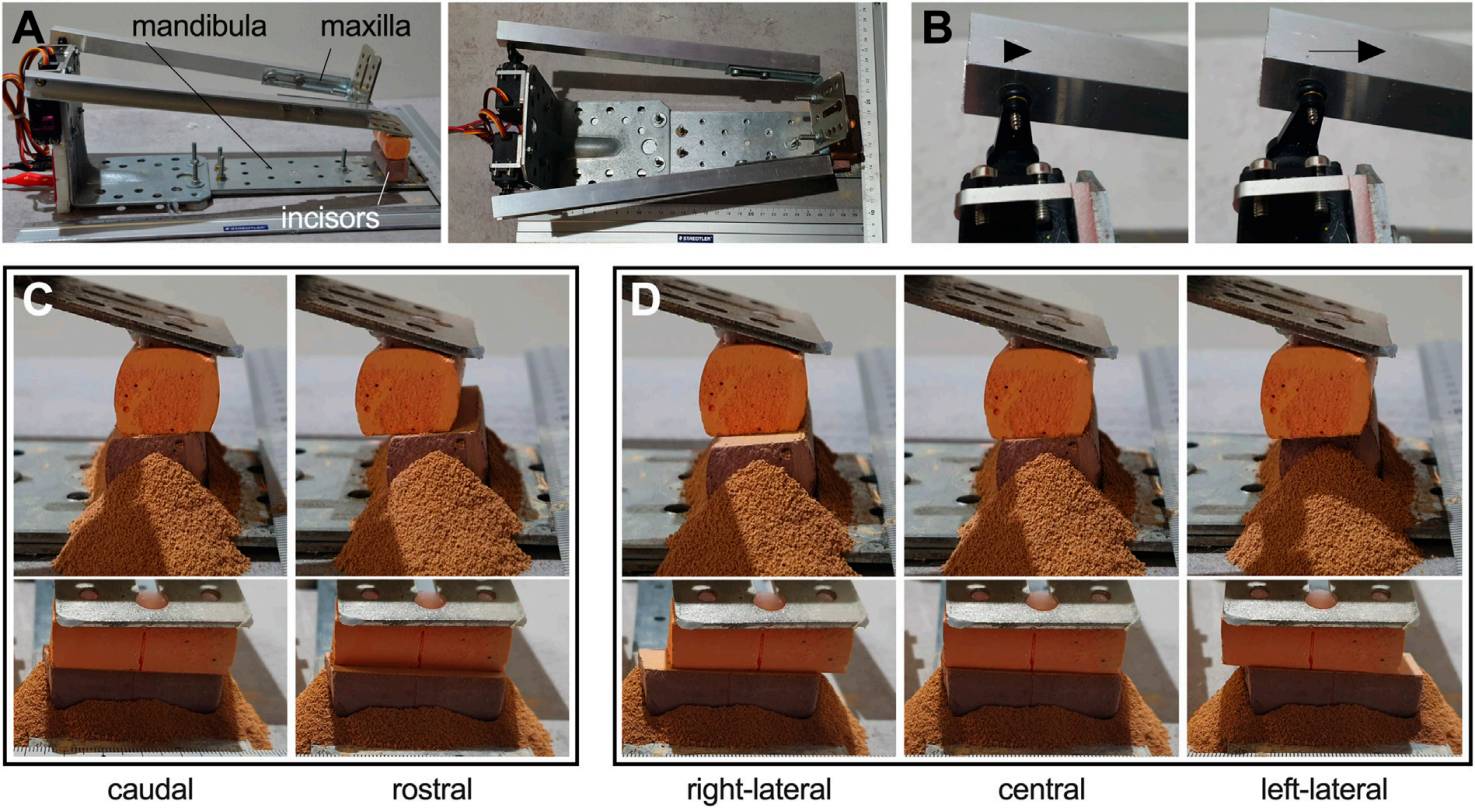
Mechanical simulator setup. **(A)** side view (left); top view (right). **(B)** example for a servo range of motion. **(C and D)** mandibular movement: **(C)** SAP– retrusion (left) and SAP+ protrusion (right). **(D)** Lateral movement from right excursion of the mandibular arcade over the neutral position to left excursion. **(C and D)** Upper panel: side view; lower panel: frontal view of the same situation. Powdery abrasion is visible around the lower arcade.

To examine the shaping of the incisor occlusal surface in relation to TMJ movements, we designed and constructed a simple mechanical simulator system (Figure 5A). The six incisor teeth were represented by chalk bars with cuboid shape and a latero-lateral width of approximately 4.75 cm, an SAP+/− extension of approximately 2.0 cm, and a low abrasion resistance. Simulator dimensions were determined to closely mimic those of the pony head used in our theoretical calculations (d_HH_ = 9.7 cm and d_HO_ = 29.1 cm). The TMJs were represented by two adjustable servo motors. As control we used an Arduino nano board (Arduino S.r.l., Italy). Rather than transferring mandibular movement to the TMJs, we emulated the assumed range of TMJ motion by designating the servomotors as the point of mandibular movement initiation. The rostro-caudal movement range of the left servomotor is illustrated in Figure 5B. Simplifying the simulator design, a mandible was rigidly connected to the test table with the upper jaw moving. To perceive the upper jaw as stationary, while the mandible moves as expected, it is necessary to move with the maxillary subordinate reference frame. The reference frame choice does not affect the results of the abrasive occlusal wear experiment. The simulator system enables mimicking TMJ movements, which in turn facilitates induction of corresponding movement patterns in the mandible during incisor occlusion (Figures 5C,D). We simulated various movement patterns, utilizing parameter sets determined in the clinical assessment. The servomotor settings are listed in Table 1.

**TABLE 1:** Tested servo motor settings to mimic TMJ movements under incisor occlusion.

Movement pattern	Assumed occlusal plane shape	Type of occlusion	SAP movement direction	Servo settings for mandibular movement	
				Laterotrusion	Protrusion–retrusion
**No. 1** (Figure 4B) symmetric to both sides	Even plane	Normocclusion	SAP+ to SAP-	±0.75 mm opposite	±1.5 mm simultaneous
			SAP⊥+ to SAP⊥-	<0.5 mm opposite	<0.75 mm simultaneous
**No. 1** (Figure 4B) asymmetric to one side	Diagonal even plane	Malocclusion	SAP+ to SAP-	±1.0 mm opposite	±1.5 mm simultaneous
			SAP⊥+ to SAP⊥-	<1.0 mm opposite	<0.75 mm simultaneous
**No. 2** (Figure 4C) symmetric to both sides	Even plane	Normocclusion	SAP+ to SAP-	±1.5 mm while other servo in rest and *vice versa*	±1.5 mm simultaneous
			SAP⊥+ to SAP⊥-	<0.75 mm for servo in motion	<0.75 mm simultaneous
**No. 2** (Figure 4C) asymmetric to one side	Diagonal even plane	Malocclusion	SAP+ to SAP-	±2.0 mm while other servo in rest	±1.5 mm simultaneous
			SAP⊥+ to SAP⊥-	<0.5 mm for servo in motion	<0.75 mm simultaneous
**No. 3**	Smile	Malocclusion	SAP+ to SAP-	<1.0 mm opposite	±1.0 mm simultaneous
			SAP⊥+ to SAP⊥-	+/− 7.0 mm opposite	±7.0 mm simultaneous
**No. 4**	Frown	Malocclusion	SAP+ to SAP-	<1.0 mm opposite	±1.0 mm simultaneous
			SAP⊥+ to SAP⊥-	−/+ 7.0 mm opposite	±7.0 mm simultaneous

SAP, sagittal angle plane.

The total number of movements was performed in 500 cycles, each consisting of 20 latero-lateral and four protrusion–retrusion movements. Values were adapted to the range of own measurements in smaller horses and theoretical assumptions.

### Data analysis and statistics

Data were analyzed using a combination of descriptive and inferential statistics. For the descriptive data analysis, measures of central tendency (i.e., mean and median) and measures of variability (i.e., standard deviation and range) were calculated for the variables of interest. We then performed normality distribution testing using a Shapiro–Wilk test to determine appropriateness using parametric tests. We also checked for outliers using the ROUT test (Q: 0.1%). The specific inferential tests used for each analysis and their outputs are described in the respective text passage or Figure caption. In addition, we performed simple linear regression analysis to examine the strength and direction of the relationship between pairs of continuous variables (age vs. OJ/CO/LE right/LE left). We used the least squares method to estimate the regression coefficients and the coefficient of determination to assess goodness of fit. The statistical significance of regression coefficients was tested using a t-distribution with a significance level of 0.05, which was used for all hypothesis testing in this study. All statistical analyses were conducted using the GraphPad Prism software package (GraphPad Software, SD, USA).

## Results

### Orthodontic measurements add to current evidence and infer no marked age dependency

**FIGURE 6:**
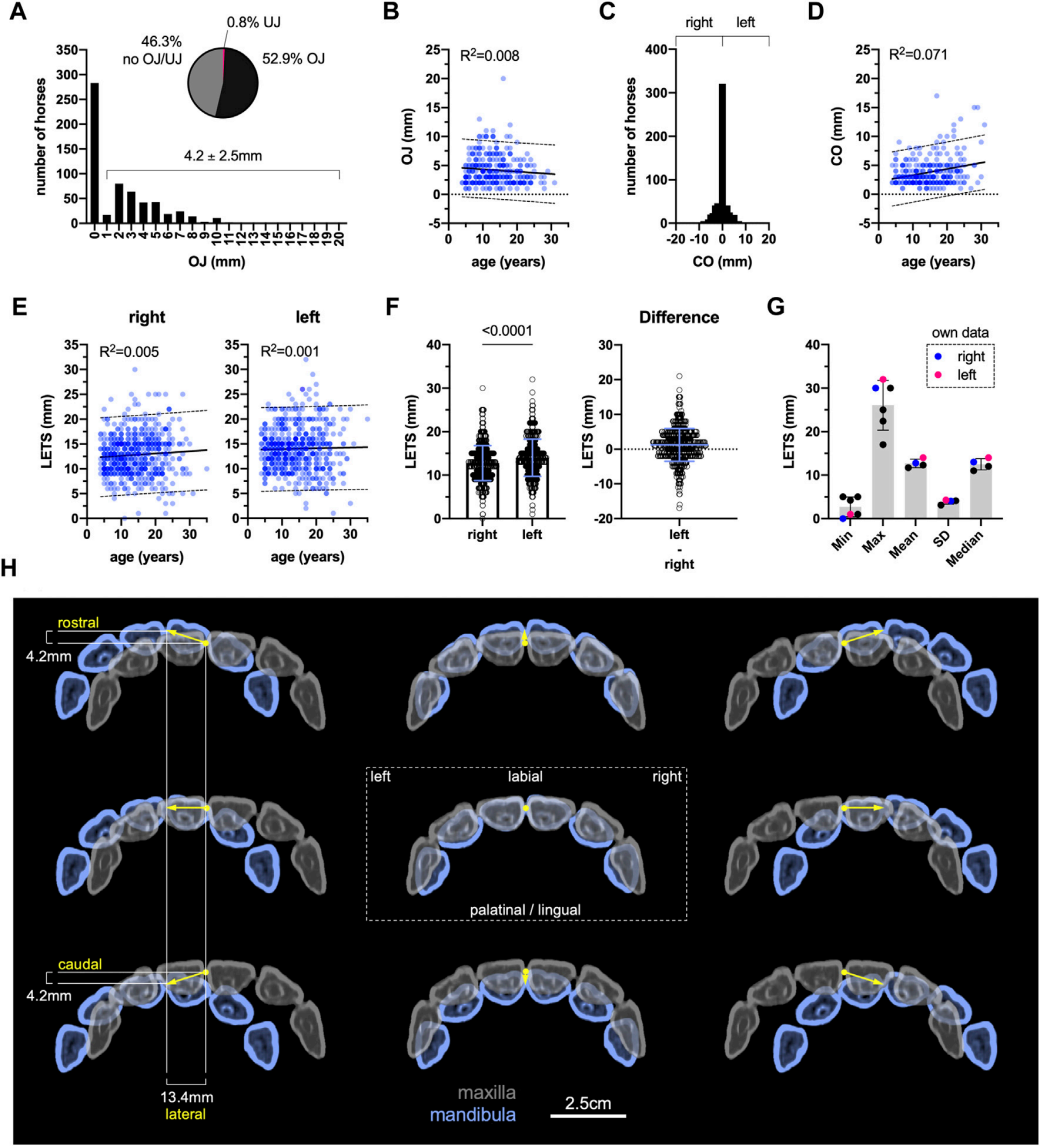
Evaluation of static and dynamic orthodontic parameters. **(A)** Overjet (OJ)/underjet absolute and relative distribution, *n =* 609. **(B)** Linear regression analysis OJ vs. age, F = 2.69, *p =* 0.102, *n =* 322. **(C)** Central incisor interdental space center offset (CO) frequency distribution and **(D)** linear regression analysis OC vs. age, F = 21.68, *p <* 0.0001, *n =* 609. **(E)** Linear regression analysis lateral excursion (LETS) to incisor separation vs. age, separated by side of jaw deflection, right: F = 2.99, *p =* 0.084; left: F = 0.31, *p* = 0.578, *n =* 604. **(F)** LETS right vs. left, two-tailed Wilcoxon matched-pairs signed rank test, *p <* 0.0001, *n =* 604. **(G)** LETS comparison to literature data. SD, standard deviation. **(H)** Data-based assumption of incisor occlusion limits.

To better approximate the range of motion that mandible and mandibular AHs in the TMJs have under incisor occlusion, we evaluated pre-treatment static and dynamic orthodontic measurements in a cohort of mainly large horses (88.2%) from South Germany. In total, data of 609 horses with a mean age (±SD) of 14 ± 6.2 years and age range from 4 to 35 years were included. In the investigated head position, OJ was the most common occlusal deviation among the horses with horizontal protrusion or retrusion of mandibular incisor teeth (Figure 6A). The population-wide mean OJ (±SD) of 2.2 ± 2.8 mm (range: 0–20 mm) excelled the extent of a mean UJ (±SD) of 0.02 ± 0.2 mm (range: 0–3 mm) both in amplitude and variation. In the underrepresented proportion of small horses (Islandic and Connemara; *n =* 55), mean OJ was 1.5 ± 2.2 mm (range: 0–10 mm), whereas UJ was not represented. In ponies (Shetland; *n =* 17), the mean OJ was 0.5 ± 1.7 mm (range: 0–7 mm), and mean UJ was 0.2 ± 0.7 mm (range: 0–3 mm). Considering only horses with OJ or UJ revealed values of 4.2 ± 2.5 mm and 2.4 ± 0.5 mm, respectively. The extent of an OJ seems not to change with an increase in age (Figure 6B).

After manual centering of the cheek teeth to symmetric position, maxillary and mandibular central incisor interdental spaces (IDS) were either located one above the other or slightly deviated to either side in most horses. A marked center offset (CO) was only observed in few horses (Figure 6C). In case of a center offset (47.3%), there was no difference between left (3.8 ± 2.7 mm) and right (3.9 ± 2.4 mm), *p =* 0.327, Mann–Whitney U test. A low proportion of variation in center offset measures can be explained by the age of horses examined, representing a weak positive association (Figure 6D).

Lateral excursion to incisor separation (LETS) to either side appears even less related to age (Figure 6E). Although mean (±SD) lateral excursion to the right (12.8 ± 4.0 mm) appeared significantly smaller than to the left (14.0 ± 4.3 mm), the overall mean difference was yet very low (Figure 6F). Comparing our overall results on lateral excursion with the values reported in the previously published literature, data are in good agreement (Figure 6G). In a small number of horses, lateral excursion to the right was 11.8 ± 4.2 mm, and that to the left was 13.2 ± 4.4 mm (range: 1–24 mm). In ponies, lateral excursion to the right and left was 8.6 ± 1.9 mm and 9.9 ± 2.8 mm, respectively (range: 4–15 mm).

These findings helped in the theoretical analyses of mandibular and TMJ angular changes and in biomechanical simulations under incisor occlusion. Figure 6H illustrates how mean overall values relate to the superposition of maxillary and mandibular six incisor teeth upon associated mandibular and TMJ movements under incisor occlusion.

### Low mandibular pitch and roll helps maintain incisor occlusal surfaces as a plane

Based on our theoretical depiction of TMJ and corresponding mandibular movement patterns during incisor occlusion, we computed the occurrence and degree of mandibular rotation Δα along a latero-lateral axis (pitch) and Δγ along the SAP+/− axis (roll). This analysis helped inferring a potential dynamic tilt of the incisor occlusal surface plane during TMJ movements under incisor occlusion. The calculations were executed for all TMJ movement patterns described (Figures 3A–E), embracing dimensions of the two horse head examples (WBL/pony).

While clinical orthodontic measurements in horses with OJ and UJ showed an average approximation to protrusion and retrusion jaw mobility of 4.2 mm and −2.4 mm respectively, we theoretically simulated these population values (mainly large horses) as the potential maximum mobility occurring in ponies. In the WBL example, we upward considered a maximum protrusion of 10 mm. Essentially, we assumed that the range of motion in our example was at the upper limit of what could be expected, while still maintaining some degree of incisor occlusal contact. Substituting $d_{⊥\mathrm{PR}}=10\;mm\;and\;d_{BO}=494\;mm$ for the WBL into Equation (5) yielded a mandibular roll of Δα ≅ 1.16 angle deg. Embracing orthodontic OJ data and pony head dimensions of our simulator resulted in a Δα<0.82 angle deg. A $d_{⊥\mathrm{PR}}=10\;mm$ and used pony head dimensions yield a slightly higher Δα of ≅ 2.0 angle deg. Computed angular deviations are alike considered not to interfere with the perception of the incisor occlusal surface as a plane.

In laterotrusion computations, movement pattern no. 1 yielded a slightly higher mandibular pitch as pattern no. 2, with a small △γ difference of 0.02 angle deg. for both the WBL and pony head (Table 2).

**TABLE 2:** Head dimensions and calculation results in laterotrusion jaw movement.

Data source		Variable	WBL	Pony
Clinical measurement		d_LETS_	13.4 mm	9.25 mm
		d_HH_	150 mm	97 mm
		d_HO_	500 mm	291 mm
		d_BO_	494 mm	287 mm
Calculation results	Movement pattern no. 1	d_AHP+L_	2.03 mm	1.56 mm
		d_AHP-R_	−2.03 mm	−1.56 mm
		△γ	1.55 deg	1.84 deg
	Movement pattern no. 2	d_AHP+L_	4.02 mm	3.08 mm
		d_AHP-R_	0.00 mm	0.00 mm
		△γ	1.53 deg	1.82 deg

Based on anatomical observations, we anticipated an immediate side shift that would involve both lateral mandibular articular head movements to be accomplished by movement components of the mandibular AHs in the same SAP⊥– direction, but of different magnitude. The angular difference △α will be of similar magnitude to △α from protrusion/retrusion movement computations, while △γ only occurs due to the difference in magnitude of equilateral SAP⊥– movement components and thus will be markedly smaller than calculated for the two rotative laterotrusion patterns.

### Simulator results confirm the normal perception of incisor occlusal surfaces as a level plane

In light of the outlined theoretical considerations, we conducted mechanical simulations of masticatory movements under incisor occlusion and analyzed the differences between various movement patterns. The results were derived after 10 k latero-lateral and 2 k retrusion–protrusion movement cycles. With reference to mean annual wear across central to corner incisor teeth in wild equids (Smuts, 1974) and what is known from domestic equine cheek teeth (Staszyk et al., 2006b; Dixon et al., 2011), we assumed an incisor wear of 2 mm/year and 1 chewing cycle per second. This led to the following: 1 cycle/sec * 3.6 k sec/h * 5 h/d * 365 days/y = 3.3 million cycles/mm in a living horse. The wear on the chalk bars in our simulator setup was around 5 mm per 10 k + 2 k cycles. Each wear simulation thus equals 2.5 years in a real horse, whereas one cycle in the simulator corresponds to 275 cycles in a living horse.

**FIGURE 7:**
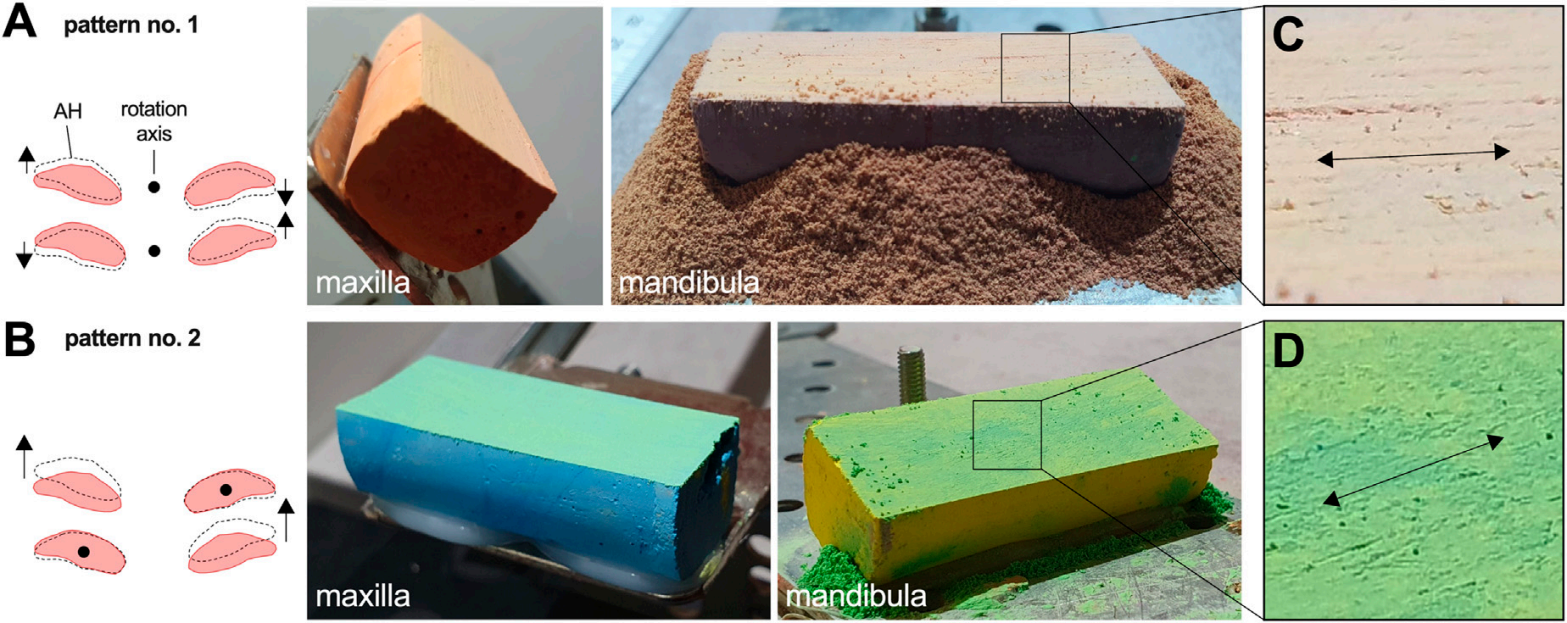
Simulation results covering symmetric latero-lateral movement patterns. Movement pattern no. 2 **(A)** and movement pattern no. 1 **(B)** both educed a flat surface plane. **(C, D)** Detail view on the chalk surface wear pattern.

Servo settings simulating the lateral jaw excursion equally with pattern no. 1 (Figure 7A), and no. 2 (Figure 7B), resulted in a flat appearance of the contact surface between chalk bars, which closely resembled the incisor occlusal surface plane in this model. For both movement patterns, the chalk bars exhibited a predominant latero-lateral surface wear pattern (Figures 7C,D).

### Asymmetric latero-lateral mandibular AH movements lead to diagonal incisor malocclusion

**FIGURE 8:**
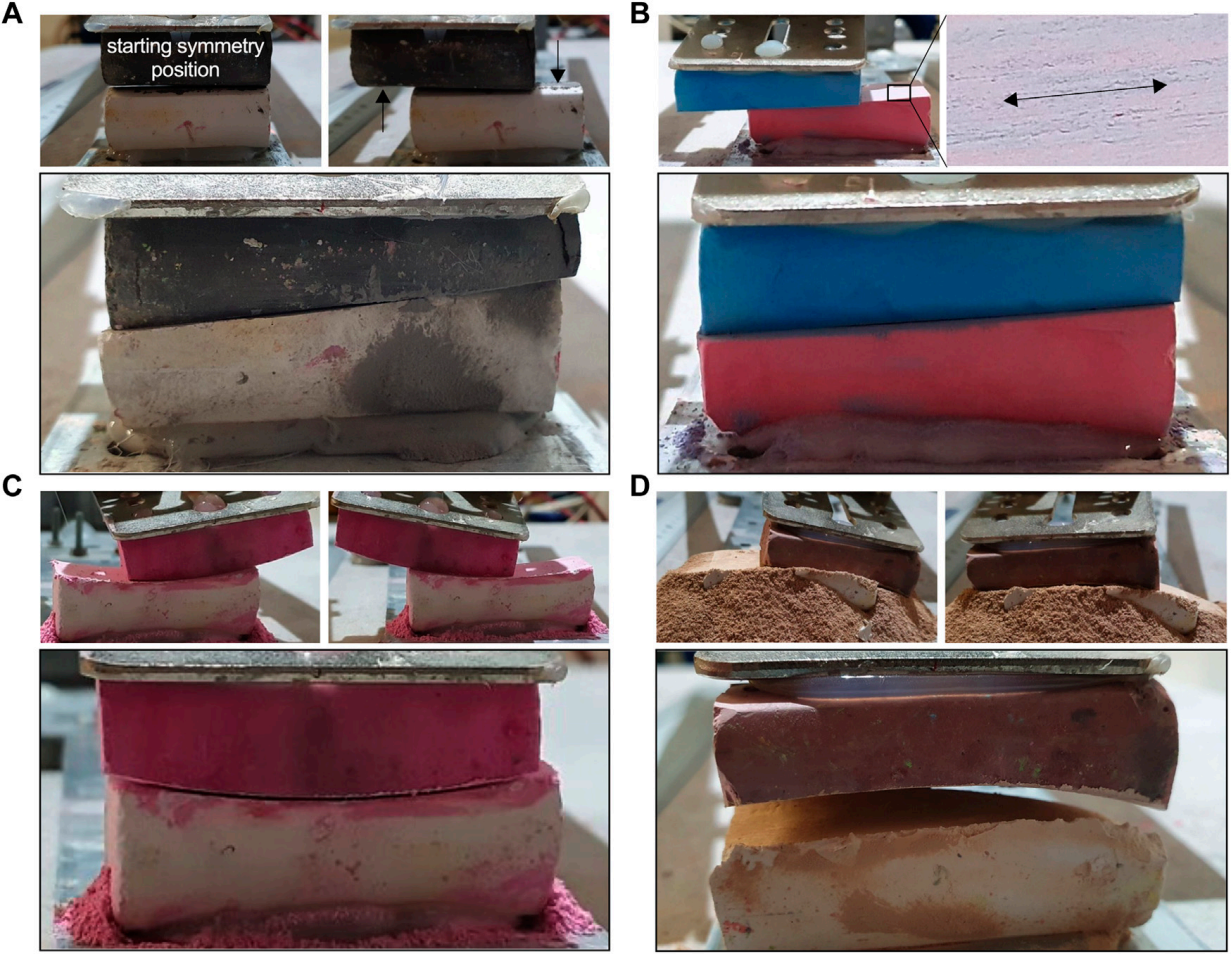
Mechanical malocclusion simulation. **(A, B)** Diagonal malocclusion representing asymmetric latero-lateral motion pattern no. 1 **(A)** and asymmetric pattern no. 2 **(B)**. Here the movement is limited by the latero-lateral symmetry position and the left point of LETS. After 20 k cycles, a clear DIM becomes visible. The black arrows in **(A)** mark the regions with reduced abrasion due to lack of contact. Despite a DIM, the occlusal surface remains largely flat and with a latero-lateral oriented wear pattern. **(C)** Smile malocclusion, **(D)** frown malocclusion.

The TMJ movement background of diagonal incisor malocclusion (DIM) development was tested transferring theoretically derived information on altered mandibular AH movements to the mechanical simulator. Servo settings were changed to run asymmetric lateral jaw excursion to the left, which was limited by the left LETS and by the symmetry position. We performed this for motion pattern no. 1 and no. 2, starting with a level plane parallel to the ground, and both simulations resulted in a flat but diagonally inclined contact surface between chalk bars (Figures 8A,B).

### Simulation of more complex patterns no. 3 and no. 4 induce smile and frown malocclusion

On the basis of laterotrusion pattern no. 1, we tested a situation where the two mandibular AH movements in SAP+ and SAP– direction are small but combined them with a large difference in left and right SAP⊥ movement components. This resulted in a pronounced mandibular roll around the SAP+/− axis with increasing excursion. Depending on whether this roll occurred in the same or in the opposite direction to the latero-lateral deflection of the mandible, a smile or frown was observed, respectively (Figures 8C,D).

## Discussion

The objective of our research was to examine the dynamic shaping of incisor occlusal surfaces in the horse. We focused on TMJ movements occurring during incisor occlusion and what influence different motion patterns have in normal incisor occlusion and malocclusion. To achieve this, we conducted clinical studies to assess the population-wide range of orthodontic parameters, including mandibular motion during incisor occlusion. Using these results, we conducted a theoretical analysis to determine the extent to which angular deviations could compromise the perception of the occlusal surface as a plane. In further sequence, clinical data and theoretically drawn assumptions were used for mechanical simulations as a proof.

During clinical dental examination, various static and dynamic orthodontic parameters such as OJ, UJ, incisor CO, LETS, DIM, smile, or frown can be assessed. Congenital malocclusion of maxillary incisor teeth that protrude labially to mandibular incisor teeth in the horizontal plane is termed overjet (OJ), while loss of occlusal contact and vertical tooth overlap is termed overbite or “parrot mouth” (Dixon et al., 2022). Similarly, labial protrusion of mandibular incisor teeth is referred to as underjet (UJ) and with vertical overlap as underbite or “monkey mouth” (Rawlinson and Earley, 2013; Dixon et al., 2022). Physiological motion dynamics, however, also need consideration when assessing these orthodontic parameters. It is known that the mandibular position relative to maxilla changes during different head positions and that there is also protrusion/retrusion mobility of the mandible during chewing (Simhofer et al., 2011; Carmalt et al., 2003; Griffin, 2013). We clinically observed that most horses included in our study exhibited a higher degree of incisor occlusal surface superimposition with lowered head position as if grazing, compared to when their head was elevated, and the mandible was retracting due to assumed passive muscle pull. It is well-described in humans that mandibular position changes with head position and is determined by muscle tone and inherent muscle visco-elasticity (Woda et al., 2001). This further leads to the assumption that state of muscle relaxation may differently affect mandibular position in sedated animals. At least to reduce head position influence on a minimum, we standardized it for orthodontic measurements. Nevertheless, describing the dynamic range of mandibular motion during incisor occlusion remains a challenge in horses. Thus, recorded OJ and UJ in our study are still an approximation, which we conservatively estimated upward in theoretical computations of mandibular pitch and roll during incisor occlusion and subsequent mechanical simulations. In the reported head position, OJ was much more frequent (52.9%) as UJ (0.8%), revealing values of 4.2 ± 2.5 mm (range: 0–20 mm) and 2.4 ± 0.5 mm (range: 0–3 mm), respectively. Domanska-Kruppa et al. (2018); Domanska-Kruppa et al. (2019) radiographically measured an OJ in 2% of 650 Warmblood foals ranging from 4 to 8 mm, but with the head fixed in a flexed position (Domanska-Kruppa et al., 2018; Domanska-Kruppa et al., 2019). Reporting an OJ prevalence of 51% in Quarter Horse foals in a head position comparable to Domanska-Kruppa et al. (2018) and Omura et al. (2015) measured mean OJ ranging from 0.5 to 1.74 mm in different age groups (Omura et al., 2015). Although reporting a similar prevalence to that observed in our study, OJ amplitude was smaller. Elevating the head to a position like that used in our study would have potentially led to higher OJ values in other studies. Gift et al. (1992) reported even higher OJ values ranging from 7.5 to 30 mm, but only evaluated horses referred due to incisor malocclusion. Dynamic protrusion/retrusion mandibular range motion during chewing was reported to be 8.8 ± 2.2 mm in larger horses (Bonin et al., 2007; Simhofer et al., 2011). This range we assumed is within the range of incisor occlusion as is our measured range of OJ upon head elevation. To even better approximate mandibular protrusion and retrusion under incisor occlusion, it is desirable in future studies to gather individual OJ or UJ in different standardized head positions.

Incisor separation upon jaw deflection in LETS measurement is a good indicator of dynamic occlusion loss in incisor teeth area and can be used adapted to head dimensions in occlusion simulation experiments. Its determination should be part of routine dental examination as it indicates the degree of cheek tooth occlusion (Easley et al., 2011b). Published mean LETS range from 11.7 to 12.3 mm (Rucker, 2002; Rucker, 2004; Pimentel and Zoppa AL do, 2014), whereas LETS in miniature horses and ponies were reported to be 5–6 mm (Rucker, 2008). Our measured population-wide mean was 14.0 ± 4.3 mm (left) and 12.8 ± 4 mm (right). The LETS measured in ponies was slightly higher in our study with 8.6 ± 1.9 mm (left) and 9.9 ± 2.8 mm (right). This may be due to reference to miniature pony breeds in literature. However, when measuring LETS, potential incisor center offset (CO) due to rostral bony or dental asymmetries needs to be considered. Such asymmetries may cause TMJ “neutral” position not to be associated with centered incisor occlusion and TMJ range of motion would likely be unaffected. However, symmetry of bilateral laterotrusion and thus incisor occlusion during mastication would be affected, resulting in development of malocclusion wear patterns such as DIM. The latter malocclusion was developed in our simulations upon mimicking asymmetric laterotrusion under incisor occlusion. If the degree of DIM in horses correlates with the extension of LETS, asymmetry needs to be further investigated on standardized clinical data.

In the theoretical description and calculations of TMJ movements, we have averted from the medical directional specifications “rostral–caudal–dorsal–ventral” since these describe possible mandibular movements in relation to the maxilla inaccurately. By using a segment coordinate system—commonly used in biomechanics (Wittenburg, 2007)—we were able to specify the directional information of the mandibular movement under incisor occlusion at the level of the occlusal surface plane accordingly. In our theoretical calculations of any possible deviations of the incisor occlusal surface from a plane, the focus was on a reasonable estimation of movement parameters causing angular deviation. The literature on dynamic masticatory biomechanics in horses is scarce (Huthmann et al., 2009; Neto et al., 2018). We therefore had to incorporate estimates of head dimensions through our own morphometric analyses on CT data sets and skull specimen. In doing so, we have always made significant upward estimates. Despite these findings, angular deviations calculated during incisor occlusion (mandibular pitch and roll) are so small that they do not affect visual perception and thus maintenance of occlusal surfaces as a plane. With a more realistic estimation of the underlying movement directions and distances, even significantly smaller angles are to be expected. As feed intake is the major motivator for chewing, it is reasonable to assume that a significant portion of tooth wear occurs not through tooth-to-tooth friction alone, but rather in conjunction with the presence of feed. The latter creates a gap between incisor and cheek tooth occlusal surfaces, resulting in additional angular changes that are hard to model. However, when incisor occlusal surfaces are subjected to chewing pressure, the distance between them should only be a few millimeters and would be mathematically assessed as the d_⊥PR_ component of mandibular AH movement. Consequently, only a small angular alteration in the sub-angle degree range can be anticipated here as well. In the worst case, this angle alteration could add up to the calculated angular change, Δα, under protrusion. Using CT-based cephalometrics in horses, Listmann et al. (2017) had shown in addition that mandibular incisor teeth under centered occlusion feature slightly steeper sagittal occlusal surface angles; mandibular = 38–44.9 angle deg.; maxillary = 32.7–35.6 angle deg. Accordingly, it can be assumed that even higher Δα than that modeled in our study (WBL = 1.16 angle deg. and pony = 0.82 angle deg.), and in addition to higher Δα due to feed intake, does not interfere with the maintenance of a flat occlusal surface plane. As a figurative representation, we found it helpful to visualize material processing with the aid of a file: only when the file is guided over a workpiece without tilting can a flat surface be achieved. When introducing high mandibular roll (Δγ) to our mechanical simulations, curvature malocclusions smile and frown are observed.

The mechanical simulator used is a very simplified representation of equine dentition. In human dental research, chewing simulators are often used for wear and fatigue tests of filling materials (Soriano-Valero et al., 2020). Several authors use a software-controlled simulator with two motor-driven axes that can mimic different motion patterns (Jensen and Abbott, 2007; Heintze et al., 2011; Rues et al., 2011; Shahin et al., 2014). Unlike a real horse, the simulator in this study has two axes of rotation, namely, the actual servo axis and the attachment point on the aluminum rail. The latter non-driven connection between the servo arm and the aluminum splint represents the TMJ hinge movement. This double joint nevertheless reproduces the SAP+/SAP– mobility with good precision. In the simulations, occlusion was maintained by gravity. All movements were thus tooth-guided, meaning under occlusion and guided by the shape of the occlusal surface. The servo drive made it possible to describe SAP+/SAP– sliding of the mandibular AH. In the horse, this movement is muscle-driven, while in the simulator the servo motor actively adjusts the AH position. Only when attempting to simulate mandibular roll around the SAP axis does a difference in the cause of AH SAP⊥ motion components occur. In the simulator, this SAP⊥ portion arose due the servo starting position choice. In the real horse, we assume the possibility of lateral movement (side shift) of the mandibular AH in addition, which, however, cannot be assessed with the current simulator in its full extent. Nevertheless, mandibular protrusion and retrusion movement in our simulator is performed on a circle segment and always comes in combination with a moderate SAP⊥ movement component as presented in Table 1. This is in alignment with our assumptions regarding TMJ movement in a horse.

Due to its rather simple construction, we used the chalk simulator only for qualitative confirmation of theoretical considerations. The focus was on achieving the planar shape of the occlusal surface plane. In addition, we were able to provide TMJ movement indicators for the development of malocclusions by deliberately changing movement patterns. In equine research, Karme et al. (2016) mounted single real maxillary and mandibular cheek teeth in a chewing machine and compared microwear and gross wear characteristics after 100,000 chewing cycles between different diets and pure attrition. The chalk bars representing incisor teeth in our study have a hardness between 1 and 2 on the Mohs hardness scale, ranging from 1 to 10. Enamel is the hardest dental tissue and ranges between 4.5 and 5.0 on the Mohs scale (Baker et al., 1959). As a result, the experimental formation of the surface shape by attrition on the occlusal surfaces in our study could already be observed after a significantly lower number of movement cycles in the order of 10 k cycles and under lower occlusal pressure of approx. 1.5 N. Englisch et al. (2018) speculated that less masticatory forces act on the incisor compared to the cheek teeth because they detected a shorter pulp to occlusal surface distance in incisor teeth. The chalk bars have the shape of a cuboid, while real arcades are more of a crescent shape. In addition, the hardness distribution in the chalk is largely homogeneous, while incisor teeth in the horse have hard enamel areas and softer dentin and cementum areas (Du Toit et al., 2008; Erickson et al., 2014). We assume, however, that the difference in shape and structure has only a marginal influence on the forming surface shape.

Horses with a DIM show a tilted incisor occlusal surface plane when viewed from the front (Rucker, 2004). Most DIMs are associated with deformation of the maxilla or incisive bone (Earley, 2011). It has also been assumed that chewing in only one direction can lead to DIM (Dixon and Dacre, 2005). The prerequisite is long-term asymmetrical mastication. In our study, a DIM could be provoked by asymmetrical laterotrusion movements, which coincides with the experience from the clinical field. Here, too, DIM is frequently reported in combination with unilateral mastication due to dental disease in the then spared cheek tooth arcade. This is consistent with the literature where skeletal deviation or asymmetry is associated with DIM (Dixon, 2002; DeLorey, 2007). Despite DIM formation, the occlusal surface hardly deviates from a flat inclined plane. Proven in our simulations, DIM formation is thus due to changed wear of the arcades resulting from asymmetric laterotrusion movements.

Equids with pronounced anisognathia tend to develop dorsal curvature of the incisor teeth (Du Toit et al., 2009). We demonstrated that a rotation of the jaw around an SAP+/SAP– axis in combination with a laterotrusion movement of the incisor teeth have the potential to explain the development of a smile or frown malocclusion. Both differ in whether the rotation and the latero-lateral movement are concurrent or opposed. In a real horse, the rotation of the jaw may be caused by the shape of the maxillary articulation area in combination with transversal mandibular AH movements. The extend of cheek tooth anisognathia could also influence the anatomically permissible degree of jaw rotation, as in pronounced anisognathia, there may be later rotation delimiting cheek tooth contact.

There is little evidence on the normal occlusal state and range in equine incisor teeth. It is important, as we did in this study here, to perform static and dynamic orthodontic measurements in a wide set of horses of different oro-dental states. We were particularly struck by lack of knowledge about the exact course of lateral jaw excursion during the chewing process as described in the introduction. Here, different explanatory models are in contradiction. On the other hand, little is known about associated TMJ movements. In high similarity to the well-understood human masticatory process, we favor laterotrusion pattern no. 2, i.e., a lateral deflection over a rotating working-side mandibular AH with simultaneous sliding of the balancing-side mandibular AH in SAP+ direction. Though, experimental confirmation would be desirable here.

## Conclusion

The study’s comprehensive findings encompassing clinical assessments, theoretical calculations, and mechanical simulations contribute to the existing body of evidence regarding orthodontic measurements, confirming the perception of the incisor occlusal surface as a plane. The clinical part provides confident delineation of the range of potential mandibular movement under incisor occlusion, leveraging LETS, and OJ or UJ measurements. This delineation holds profound implications for theoretical appraisal of dynamic angular changes in the mandible and TMJs as well as biomechanical simulations. Intriguingly, our study introduces a novel perspective by introducing the segmental coordinate system to the equine head, shedding fresh light on the intricacies behind the formation of a level occlusal surface plane. By amalgamating analyses of possible laterotrusion movement patterns no. 1 and no. 2, we systematically ascertain angular deviations. The insights from this study serve as a foundational cornerstone, facilitating further explorations and potentially leading to invaluable advancements in dental care of horses through the application of orthodontic measurements.

We hope that our study will contribute to early identification of further dentition abnormalities and allow timely intervention and prevention. Overall, it could support clinicians in diagnosing and managing dental conditions more effectively by applying more targeted and individualized treatment strategies for the sake of an improved oral health and wellbeing of the horse.

## Study limitations

First, our modeling relies on physiologic horse dentition and does not incorporate variables such as cheek tooth pathologies, dental treatment, and feed consistency, which may influence the mandibular range of motion and incisor attrition. Second, the qualitative results obtained from mechanical simulations limit our ability to draw quantitative conclusions due to the system’s simplicity and expected deviations from real-world scenarios. Simulator design mainly limits the accurate representation of side shift movement. These limitations should be taken into consideration when interpreting the findings of this study.

## Data Availability

The raw data supporting the conclusions of this article will be made available by the authors, without undue reservation.
